# Short-term progression of cardiometabolic risk factors in relation to age at type 2 diabetes diagnosis: a longitudinal observational study of 100,606 individuals from the Swedish National Diabetes Register

**DOI:** 10.1007/s00125-017-4532-8

**Published:** 2018-01-09

**Authors:** Andri O. Steinarsson, Araz Rawshani, Soffia Gudbjörnsdottir, Stefan Franzén, Ann-Marie Svensson, Naveed Sattar

**Affiliations:** 1 School of Health Science, Faculty of Medicine, University of Iceland, Reykjavík, Iceland; 2 Department of Molecular and Clinical Medicine, Institute of Medicine, University of Gothenburg, Gothenburg, Sweden; 3 The Swedish National Diabetes Register, Västra Götalandsregionen, Gothenburg, Sweden; 4 BHF Glasgow Cardiovascular Research Centre, Institute of Cardiovascular and Medical Sciences, University of Glasgow, 126 University Place, Glasgow, G12 8TA UK

**Keywords:** Age group, Blood glucose, BMI, Cardiometabolic risk, Cardiovascular disease, Lipids, Premature mortality, Type 2 diabetes

## Abstract

**Aims/hypothesis:**

The reasons underlying a greater association of premature mortality with early-onset type 2 diabetes relative to late-onset disease are unclear. We evaluated the clinical characteristics at type 2 diabetes diagnosis and the broad trajectories in cardiometabolic risk factors over the initial years following diagnosis in relation to age at diagnosis.

**Methods:**

Our cohort consisted of 100,606 individuals with newly diagnosed type 2 diabetes enrolled in the Swedish National Diabetes Register from 2002 to 2012. The average follow-up time was 2.8 years. Analyses were performed using a linear mixed-effects model for continuous risk factors and a mixed generalised linear model with a logistic link function for dichotomous risk factors.

**Results:**

The individuals diagnosed at the youngest age (18–44 years) were more often male and had the highest BMI (mean of 33.4 kg/m^2^) at diagnosis and during follow-up compared with all other groups (those diagnosed at 45–59 years, 60–74 years and ≥75 years; *p* < 0.05), being ~5 kg/m^2^ higher than the oldest group. Although HbA_1c_ patterns were similar between all age groups, there was a difference of about 5 mmol/mol (0.45%) between the two groups at 8 years post-diagnosis (*p* < 0.05). Additionally, individuals diagnosed younger had ~0.7 mmol/l higher triacylglycerol, and ~0.2 mmol/l lower HDL-cholesterol levels at diagnosis relative to the oldest group. Such differences continued for several years post diagnosis. Yet, although more of these younger individuals were receiving oral glucose-lowering agents, other cardioprotective therapies were prescribed less often in this group. Differences in BMI, blood glucose and lipid levels remained with adjustment for potential confounders, including marital status, education and country of birth, and, where relevant, differential treatments by age, and in those with at least 5 years of follow-up.

**Conclusions/interpretation:**

Individuals who develop type 2 diabetes at a younger age are more frequently obese, display a more adverse lipid profile, have higher HbA_1c_ and a faster deterioration in glycaemic control compared with individuals who develop diabetes later in life. These differences largely remain for several years after diagnosis and support the notion that early-onset type 2 diabetes may be a more pathogenic condition than late-onset disease.

**Electronic supplementary material:**

The online version of this article (10.1007/s00125-017-4532-8) contains peer-reviewed but unedited supplementary material, which is available to authorised users.


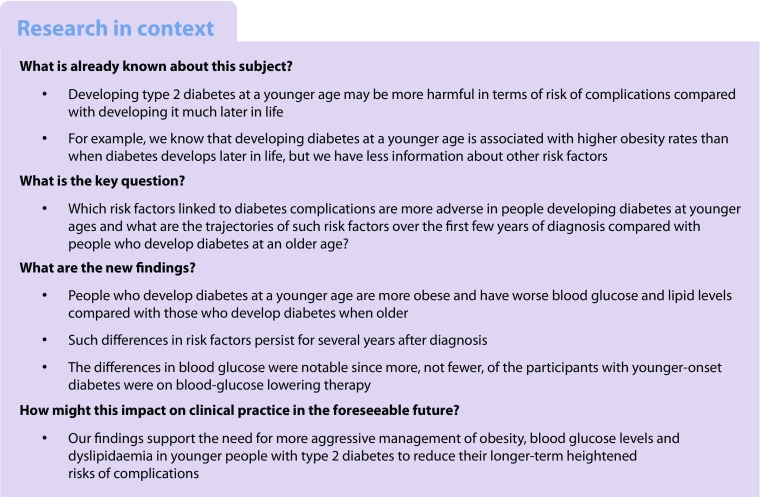


## Introduction

The prevalence of diabetes mellitus has been increasing during the past few decades with rapid rises in incidence in the 1990s and early 2000s [1, 2]. Type 2 diabetes is now more frequently diagnosed in young adults and adolescents [3–5]. The obvious reason for this is increasing obesity among young people. In the 1990s, obesity increased by 70% in people aged 18–29 years in the USA and, in the same decade, type 2 diabetes also increased by 70% in people aged 30–39 years [2, 6]. Consequently, in recent years, type 2 diabetes has sometimes been categorised as early-onset diabetes, i.e. people diagnosed before the age of 45, and later-onset (or usual-onset), i.e. people diagnosed ≥45 years of age [7].

Cardiovascular disease (CVD) is a major cause of mortality in individuals with type 2 diabetes [8, 9]. From a cardiovascular perspective, early-onset type 2 diabetes appears to be more aggressive than later-onset disease. For example, when younger people are diagnosed with type 2 diabetes they often already have several risk factors for developing CVD [10–12]. Our research group recently confirmed that younger age at type 2 diabetes diagnosis and poor glycaemic control were individually correlated with excess risk of death from any cause and from CVD [13]. Furthermore, others have shown that early-onset type 2 diabetes is more strongly pathogenic than type 1 diabetes [14]. However, the reasons behind the higher risk of complications in early-onset type 2 diabetes are not fully understood.

Whilst there are some data to suggest that obesity and differential changes in blood glucose may be operating in early-onset type 2 diabetes, a comprehensive study examining major CVD risk factors according to age at diagnosis, as well as examining subsequent trends in risk factor control, is lacking. Therefore, the aims of this study were to evaluate, in relation to age at diagnosis: (1) clinical characteristics at diagnosis; (2) trajectories (time trends) in CVD risk factors over the initial years following type 2 diabetes diagnosis; and (3) whether any apparent differences were inter-related (e.g. BMI vs lipids) or could be easily explained by differential treatments (e.g. statins and glucose-lowering drugs).

## Methods

The primary aim of this observational study was to describe how CVD risk factors change over time from diagnosis of type 2 diabetes depending on age at diagnosis in using data from the Swedish National Diabetes Register (NDR). The NDR was launched in 1996 as a nationwide clinical quality register that aims to support and monitor improvements in the quality of diabetes care. It now contains clinical data on about 90% of individuals aged ≥18 years with diabetes (type 1 and type 2) in Sweden. Before inclusion into the NDR informed consent has to be provided by each participant [15, 16]. Ethical approval to conduct this study was acquired from the Regional Ethical Review Board in Gothenburg, Sweden.

### Study population

Individuals with newly diagnosed type 2 diabetes who had at least one listing in the NDR between 1 January 2002 and 31 December 2012 were identified. Type 2 diabetes was defined on the basis of epidemiological data: individuals receiving dietary treatment only, oral glucose-lowering agents only, or those diagnosed at ≥40 years of age receiving insulin therapy or insulin and oral glucose-lowering agents. In Sweden, the WHO diagnostic criteria are used to diagnose diabetes [17]. All individuals who did not have information on age at diagnosis of diabetes were excluded. We assessed potential misclassification bias by comparing the concordance between the epidemiological definitions of type 2 diabetes with the clinician’s classification of diabetes type (which is also available in the NDR); we confirmed that 97% of our cohort were also classified as type 2 diabetes by their clinician.

Newly diagnosed individuals in each age category (age at type 2 diabetes diagnosis) were followed until death or end of follow-up on 31 December 2012. The median follow-up was 2.22 years (interquartile range, 0.80–4.31 years; maximum, 10.9 years; mean follow-up was 2.8 years). Thus, we studied individuals who were enrolled in the NDR in the same year that they were diagnosed and we followed them for up to 10 years. Reporting into the NDR is performed approximately annually, which results in multiple measurements being available for each participant. Our final cohort consisted of 100,606 individuals contributing 679,420 observations. We categorised the cohort into the following four age groups (age at type 2 diabetes diagnosis was equal to actual age owing to the inclusion criteria): 18–44 years, 45–59 years, 60–74 years and ≥75 years.

### Details on variables

Glycaemic control was estimated based on HbA_1c_ measurements, which were calibrated nationwide with the HPLC Mono-S method and converted into mmol/mol according to the International Federation of Clinical Chemistry [18]. Systolic BP (SBP) and diastolic BP (DBP) were measured in mmHg. BMI was calculated as weight (kg) divided by the height squared (m^2^). Blood lipid profile was estimated based on LDL-cholesterol, HDL-cholesterol, triacylglycerols and total cholesterol measurements, all measured in mmol/l. Serum creatinine was measured in μmol/l and eGFR was carried out by the Modification of Diet in Renal Disease (MDRD) study equation.

Microalbuminuria was defined as two positive results for three samples obtained within 1 year of each other, with an albumin/creatinine ratio of 3–30 mg/mmol or a urinary albumin clearance of 20–200 μg/min. All variables were assessed from 2002 to 2012. Initial medication data were obtained within 3 months of diagnosis.

### Statistical analysis

Continuous dependent variables (e.g. SBP) were analysed using a mixed-effects model that incorporated a random participant effect, fixed effects of age group, time, and a time by age group interaction where time was a categorical variable. This allowed for separate estimates of time trends for each age group. HbA_1c_ observations were analysed starting 1 year after diagnosis to avoid the rapid drop in HbA_1c_ commonly seen during the first year. The models contained adjustment factors, such as sex, BMI, smoking and concomitant treatment subject to convergence, as well as, where relevant, socioeconomic (marital status and education) measures and country of birth (categorised as in Table 1). Complete information on other adjustment variables is available in the ESM Table 1. The mixed-repeated-measures model handles missing data under the assumption of missing at random without relying on crude imputation techniques, such as last value carried forward, and is therefore less prone to bias.

**Table 1.** Baseline descriptive statistics stratified by age groups.

Variable	Missing data, *n* (%)	Overall	18–44 years of age	45–59 years of age	60–74 years of age	≥75 years of age
*n*		100,606	8642	30,907	45,269	15,788
Follow-up time (years)		2.8 (2.5)	2.5 (2.5)	2.9 (2.6)	2.9 (2.5)	2.7 (2.3)
Sex, women	0 (0.0)	44,925 (44.7)	3442 (39.8)	11,695 (37.8)	19,865 (43.9)	9923 (62.9)
Age (years)	0 (0.0)	62.0 (12.3)	38.3 (5.6)	53.1 (4.2)	66.2 (4.1)	80.4 (4.4)
HbA_1c_	8208 (8.2)					
mmol/mol		49.2 (11.0)	51.5 (14.6)	50.4 (12.3)	48.2 (9.7)	48.5 (9.4)
%		6.7 (1.0)	6.9 (1.3)	6.8 (1.1)	6.6 (0.9)	6.6 (0.9)
>53 mmol/mol		16, 462 (17.6)	1782 (32.4)	6001 (28.0)	6360 (20.0)	2319 (20.8)
BMI (kg/m^2^)	25,701 (25.5)	30.6 (5.7)	33.4 (7.3)	31.5 (5.8)	30.2 (5.2)	28.3 (4.7)
BMI >30 kg/m^2^	25,701 (25.5)	36,437 (48.6)	4131 (65.3)	12,964 (55.7)	15,753 (46.3)	3589 (31.9)
SBP (mmHg)	18,480 (18.4)	137.3 (17.3)	128.4 (15.3)	134.9 (16.5)	139.1 (16.9)	141.6 (18.3)
Systolic hypertension (>140 mmHg)	18,480 (18.4)	26,766 (32.6)	982 (14.8)	6737 (26.9)	13,502 (36.2)	5545 (42.0)
DBP (mmHg)	18,480 (18.4)	79.7 (10.0)	80.5 (10.5)	82.0 (9.9)	79.4 (9.6)	75.6 (9.8)
Diastolic hypertension (>90 mmHg)	18,480 (18.4)	7918 (9.6)	809 (12.2)	3494 (14.0)	3109 (8.3)	506 (3.8)
Total cholesterol (mmol/l)	33,648 (33.4)	5.3 (1.2)	5.3 (1.2)	5.4 (1.2)	5.2 (1.1)	5.1 (1.1)
Hypercholesterolaemia (>6.2 mmol/l)	33,648 (33.4)	12,208 (18.2)	939 (17.7)	4401 (20.8)	5519 (17.6)	1349 (14.8)
LDL-cholesterol (mmol/l)	42,691 (42.4)	3.1 (1.0)	3.2 (1.0)	3.2 (1.0)	3.1 (1.0)	3.0 (1.0)
HDL-cholesterol (mmol/l)	40,893 (40.6)	1.2 (0.4)	1.1 (0.3)	1.2 (0.4)	1.3 (0.4)	1.3 (0.4)
Triacylglycerol (mmol/l)	40,037 (39.8)	2.0 (1.4)	2.4 (1.8)	2.2 (1.6)	1.9 (1.2)	1.7 (0.9)
Microalbuminuria^a^	51,166 (50.9)	6110 (12.4)	492 (11.6)	1820 (11.6)	2732 (12.2)	1066 (15.1)
eGFR (ml/min)	22,951 (22.8)	84.8 (25.2)	105.8 (26.9)	93.5 (25.3)	81.8 (21.1)	67.0 (19.9)
Smoker	22,205 (22.1)	14,257 (18.2)	1540 (24.1)	6117 (25.2)	5875 (16.5)	725 (5.9)
Medication use^b^						
Antihypertensive agents	7845 (7.8)	57,686 (62.2)	1801 (23.1)	14,499 (51.2)	29,630 (70.5)	11,756 (80.4)
Statins	7927 (7.9)	35,582 (38.4)	1285 (16.3)	9575 (33.6)	19,029 (45.4)	5693 (39.5)
No medication	0 (0.0)	52,491 (52.2)	3824 (44.2)	14,094 (45.6)	24,533 (54.2)	10,040 (63.6)
Oral glucose-lowering agents	0 (0.0)	38,582 (38.3)	3973 (46.0)	13,197 (42.7)	16,894 (37.3)	4518 (28.6)
Insulin	0 (0.0)	5589 (5.6)	525 (6.1)	2031 (6.6)	2194 (4.8)	839 (5.3)
Insulin + oral glucose-lowering agents	0 (0.0)	3944 (3.9)	320 (3.7)	1585 (5.1)	1648 (3.6)	391 (2.5)
Marital status	448 (0.4)					
Single		18,283 (18.3)	4099 (47.5)	8155 (26.5)	5212 (11.6)	817 (5.2)
Married		53,276 (53.2)	3587 (41.6)	15,933 (51.7)	26,678 (59.2)	7078 (45.3)
Separated		17,917 (17.9)	910 (10.5)	6194 (20.1)	9042 (20.1)	1771 (11.3)
Widowed		10,682 (10.7)	35 (0.4)	549 (1.8)	4152 (9.2)	5946 (38.1)
Country of birth	0 (0.0)					
Sweden		81,545 (81.1)	5839 (67.6)	23,267 (75.3)	38,412 (84.9)	14,027 (88.8)
Europe (excl. Sweden)		10,612 (10.5)	710 (8.2)	3441 (11.1)	5047 (11.1)	1414 (9.0)
Rest of world		8449 (8.4)	2093 (24.2)	4199 (13.6)	1810 (4.0)	347 (2.2)
Education	1881 (1.9)					
Elementary school		36,743 (37.2)	1881 (22.2)	8099 (26.5)	17,687 (39.8)	9076 (59.6)
College level		44,029 (44.6)	4719 (55.8)	15,862 (51.9)	18,939 (42.6)	4509 (29.6)
Upper/secondary school		17,953 (18.2)	1858 (22.0)	6617 (21.6)	7827 (17.6)	1651 (10.8)

All data are based on the first observation after diagnosis, except for HbA_1c_ where the first observation 1 year after diagnosis was used

All continuous variables are represented as mean (SD). Dichotomous variables are represented as number of individuals (%).

^a^Microalbuminuria was defined as two positive results for three samples obtained within 1 year of each other, with an albumin/creatinine ratio of 3–30 mg/mmol or a urinary albumin clearance of 20–200 μg/min

^b^Initial medication data were obtained within 3 months of diagnosis

The graphical representation of the data is based on estimated and observed yearly averages with 95% CI for up to 8 years after diagnosis. Sensitivity analyses, adjusted for confounders, were conducted for individuals with at least 5 years of follow-up (ESM Fig. 2a–e), which included adjustments for the confounders shown in ESM Table 1. Further sensitivity analyses were additionally adjusted for marital status, education and country of birth.

A *p* value <0.05 was considered to be statistically significant and, since no adjustment for multiple comparisons has been made, the interpretations should focus on the overall patterns rather than the outcomes of single hypothesis tests. All statistical analyses were conducted with SAS (version 9.4; www.sas.com/en_gb/software/sas9.html, accessed 10 November 2017) and R (version 3.2.3) (https://www.rstudio.com/products/rstudio/download/; https://cran.hafro.is/; both accessed 10 November 2017).

## Results

### Baseline characteristics and risk factor differences

Table 1 shows baseline characteristics in the overall cohort stratified by age group. Baseline was defined as the participant’s first observation in the NDR at year of diagnosis. Women represented 44.7% of the total cohort. The mean age (years) in each age group was 38.3, 53.1, 66.2 and 80.4. At diagnosis, the youngest group contained proportionately more men, had a higher mean BMI by about 5 kg/m^2^ and a higher mean HbA_1c_ of ~3 mmol/mol (0.27%) compared with the oldest group. The youngest group also had the highest triacylglycerol and the lowest HDL-cholesterol levels of all the groups. However, this group had the lowest SBP and one of the highest DBP compared with the other age groups. In terms of treatments, statin use was lowest in youngest age group, being almost three times lower than rates of use in the 60–74 years age group. In contrast, oral glucose-lowering agent use was higher in the youngest group at 46% vs 29% in the oldest group. In addition, antihypertensive agent use was considerably lower in the youngest group at 23% vs 80% in the oldest group. Considerably more of the younger cohort were from countries outside Europe. Men had higher HbA_1c_ levels at diagnosis compared with women, but women had higher BMI (ESM Table 2).

### Risk factor trajectories

**Fig. 1:**
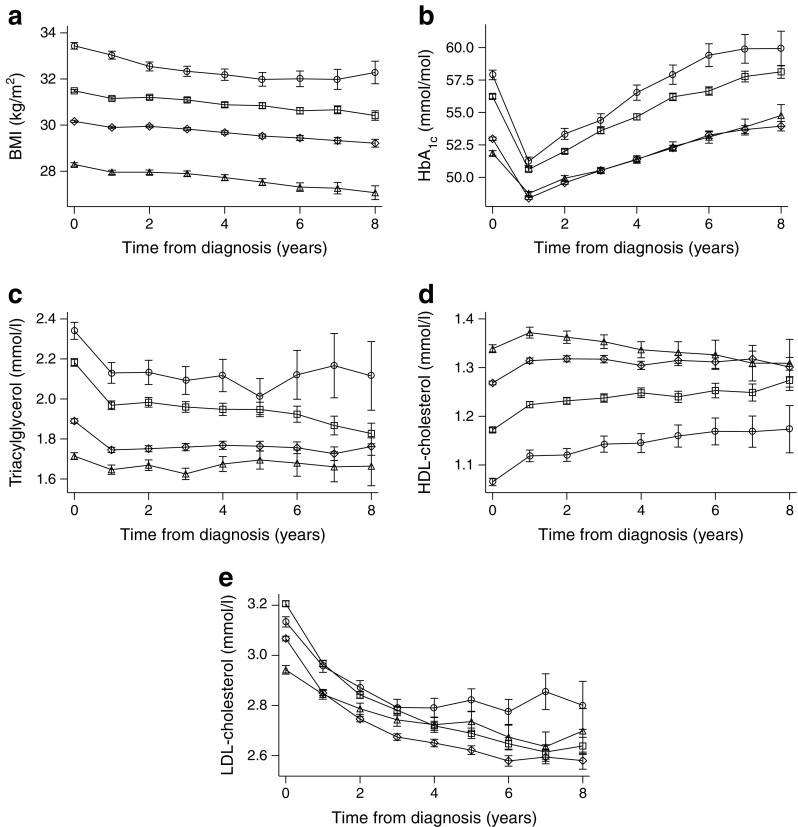
Yearly averages (95% CI) stratified by age group for (**a**) BMI, (**b**) HbA_1c_, (**c**) triacylglycerol, (**d**) HDL-cholesterol and (**e**) LDL-cholesterol. White circles, 18–44 years old; plus sign (+), 45–59 years old; crosses (×), 60–74 years old; white triangles, ≥75 years old. All analyses, *p* < 0.05 where 95% CI do not overlap

The trajectories of cardiometabolic risk factors over time by age at type 2 diabetes diagnosis are presented in Fig. 1. The significantly (*p* < 0.05) higher BMI level observed in the youngest age group at diagnosis was sustained over time relative to the other groups (Fig. 1a). A widening and significant (*p* < 0.05) difference in HbA_1c_ was observed over time, with a difference of ~5 mmol/mol (0.45%) at 8 years between the youngest vs two oldest age groups (Fig. 1b).

Lipid levels remained significantly (*p* < 0.05) different between age groups, with the youngest group showing continually higher triacylglycerol levels (by ~0.5–0.6 mmol/l) compared with the two oldest age groups, and lower HDL-cholesterol levels compared with all other age groups (although this latter difference narrowed over time) (Fig. 1c,d). The differences in LDL-cholesterol levels between age groups widened over time; even though levels in all groups declined this widening probably reflects more frequent use of statins in the older age groups (Fig. 1e).

In summary, individuals that developed type 2 diabetes at a younger age had greater aggregated exposure (i.e. AUC) to higher BMI, higher blood glucose levels and abnormal lipids compared with those who developed diabetes at an older age.

Other risk factors (including BP, renal function, total cholesterol and albuminuria) also changed over time (ESM Fig. 1a–e), but most were as expected. The use of statins and antihypertensive agents was lowest in the youngest age group at diagnosis and this was sustained, at least in the early years following diagnosis (ESM Fig. 1f,g). Finally, number of current smokers was highest in the youngest age group and this did not change over time (ESM Fig. 1h).

### Sensitivity analyses in individuals with at least 5 years follow-up

When we restricted analyses to only include individuals with at least 5 years follow-up and adjusted for key confounders for each of the main risk factors as depicted in ESM Table 1, the findings were broadly similar to those reported for the entire cohort with unadjusted analyses (see ESM Fig. 2a–e). For example, the trajectory of blood glucose levels after the first year was significantly (*p* < 0.05) greater in the youngest group relative to the older groups, leading to the highest blood glucose levels observed in this group at year 6, despite adjustment for diabetes medication and BMI. Likewise, differences in lipid abnormalities persisted despite adjustment for sex, BMI and lipid-lowering therapy. Further analyses adjusting for marital status, education and country of birth did not materially change the pattern of results (ESM Fig. 3a–e).

## Discussion

To the best of our knowledge, this study of 100,606 individuals with newly diagnosed type 2 diabetes is the largest and perhaps the first to compare in detail trajectories on several cardiometabolic risk factors between individuals with type 2 diabetes grouped according to age of diagnosis in a European (white) population, complementing and extending data from a recent study in China [19]. The results from our study show that age at diagnosis of type 2 diabetes is an important aspect of risk factor control with regard to adiposity, blood glucose and lipid levels; these factors were all worse in individuals diagnosed at a younger age and throughout the early years following diagnosis, during which the use of glucose-lowering medication was higher in this group relative to older individuals. Moreover, such differences appear not to be accounted for by obvious confounders including differential drug therapy rates, sex, smoking and, where relevant, BMI differences by age group. The results were also maintained when we adjusted for socioeconomic variables and, in particular, country of birth. Therefore, our findings support the notion that early-onset type 2 diabetes may be a more pathogenic condition per se than later-onset type 2 diabetes, helping to explain the observation of greater life-years lost when diabetes is diagnosed at a younger age, as recently reported [20, 21].

Our data on the relationship between age at diagnosis and cardiometabolic risk factors meaningfully extend previous studies [22, 23] as it is contemporary and multiple-fold larger in terms of size. Our results also hold potential clinical relevance. With respect to hyperglycaemia, despite a higher rate of glucose-lowering agent use in younger (i.e. ≤45 years of age) compared with older individuals (Table 1), younger individuals had higher HbA_1c_ levels and worse trajectories over time, so their aggregated exposure to hyperglycaemia is worse from the point of diagnosis (and potentially prior to diagnosis if diagnosed later). Therefore, total exposure to hyperglycaemia is clearly higher in younger individuals and, since hyperglycaemia is a strong and independent predictor for microvascular complications and has long-term consequences for CVD in individuals with type 2 diabetes [13, 24, 25], the risk of complications driven in part by hyperglycaemia should be higher in this group. Younger individuals may experience delayed diabetes diagnoses owing to a lower likelihood of opportunistic screening, but the poorer HbA_1c_ trajectories observed in this group despite similar or greater prescription rates for glucose-lowering medications suggest that undertreatment is unlikely to be the only reason for poor glycaemic control over time. Clearly, younger individuals are more frequently obese and their aggregated BMI over time is higher than in older individuals, which is possibly related to higher blood glucose levels over time; however, our sensitivity analyses suggest that blood glucose levels remain higher in the younger group even with adjustment for BMI (ESM Fig. 2b) and with adjustment for other factors including country of birth (ESM Fig. 3b). Of course, genetic factors may also be of greater importance for early-onset type 2 diabetes.

Dyslipidaemia, characterised by low HDL-cholesterol and elevated triacylglycerol levels, is a risk factor for CVD [25]. In this study, younger individuals had lower HDL-cholesterol levels and higher triacylglycerol levels at diagnosis, in accordance with most previous studies [22, 23, 26]; although Hillier et al, in a much smaller study, did not observe a difference in triacylglycerol levels between individuals with early-onset and later-onset type 2 diabetes [23]. Whilst recent evidence indicates that higher triacylglycerol levels are causally linked to CVD [27], other strong evidence indicates earlier hyperlipidaemia (as measured by either LDL-cholesterol or non-HDL-cholesterol—also higher in younger individuals in the present study) is associated with long-term harm [28]. These collective findings broadly concur with greater obesity in younger individuals, although, once again, lipid differences remained with adjustments for BMI and lipid-lowering therapy use, which was lower in younger individuals in the early years following diagnosis and did not catch up with older groups for at least several years. This is notable since these younger individuals may have more to profit from preventive treatments, such as statins, in terms of gain of life-years free from CVD events [29].

The poorer BMI, HbA_1c_ and lipid profiles observed in the younger individuals helps to explain why early-onset type 2 diabetes may confer a very high lifetime risk of CVD and premature death compared with later-onset disease [13, 19, 30]. It is clear that the relative hazard of developing CVD is much greater in individuals with early-onset than later-onset disease, compared with age-matched individuals without diabetes [7]. There have been increasing calls [29, 31] for earlier treatment of cardiometabolic risk factors for individuals with high lifetime risks of CVD to enhance survival benefits; younger individuals with type 2 diabetes fit these criteria well.

The strength of this study is that it is a nationwide study which included virtually all individuals with newly diagnosed type 2 diabetes in Sweden within the study period, and is thus representative of the general diabetes population. This is in contrast to smaller observational studies. However, we fully accept some, often inevitable, limitations. For example, our study only included Swedish nationals; whilst our results should be relevant to many high-income countries with high proportions of individuals of European descent, our work needs to be replicated in other ethnic groups. We also recognise that the overall mean length of follow-up was modest, but note that the same trends were observed in individuals who had at least 5 years of follow-up (ESM Fig. 2a–e). In addition, we cannot rule out the probability of missing BMI values from individuals with normal or close to normal BMI because these individuals may be less likely to have their BMI recorded, which could lead to a potential bias that would in fact underestimate, rather than overestimate, diagnosis-related BMI differentials by age, as well as changes in BMI over time. However, our BMI data fit well with recent findings in a Scottish diabetes dataset, lending some external validity [32]. Finally, we acknowledge that higher HbA_1c_ levels at diagnosis in younger individuals is due, in part, to a greater delay in diagnosis compared with older individuals, perhaps because younger individuals are less likely to be captured by opportunistic screening. However, blood glucose levels remained higher over time, even after diagnosis, in those under 45 years of age (despite more of this group being on glucose-lowering therapy), potentially suggesting some innate contribution to higher risk blood glucose profiles.

In summary, individuals with early-onset type 2 diabetes are more obese at diagnosis and for several years afterwards, have higher blood glucose levels, and a faster deterioration of glycaemic control following 1 year of diagnosis compared with individuals diagnosed at an older age. They also have more atherogenic lipid profiles. Since such differences remained with adjustment for obvious confounders, including, where relevant, glucose- or lipid-lowering treatments and country of birth, our results support the notion that early-onset type 2 diabetes is a more pathogenic condition than later-onset disease. Irrespective of the reasons for worse adiposity, lipid and glycaemic risk factor profiles in the younger age group, these real-life data suggest a need for more aggressive management of type 2 diabetes in younger individuals to lessen life-years lost in this high-risk group.

## Electronic supplementary material

ESM (PDF 940 kb)

## Data Availability

The data that support the findings of this study are available from the corresponding author in anonymised form upon reasonable request.

## Abbreviations

CVD: Cardiovascular disease
DBP: Diastolic BP
NDR: The Swedish National Diabetes Register
SBP: Systolic BP
