# Supplementary material for: Short-term progression of cardiometabolic risk factors in relation to age at type 2 diabetes diagnosis: a longitudinal observational study of 100,606 individuals from the Swedish National Diabetes Register
Source: Diabetologia. 2018 Jan 9;61(3):599–606. doi: 10.1007/s00125-017-4532-8 (PMC6448968; doi:10.1007/s00125-017-4532-8)
Supplement: Supplementary file 1 — (PDF 940 kb) [file 125_2017_4532_MOESM1_ESM.pdf]

Electronic Supplementary Material (ESM)

ESM Table 1: Further factors adjusted for in those with five years' follow-up (ESM Figures 2a-e below).

**Outcome**	**Model adjustment variables**
HbA_{1c}	Sex, BMI, Smoking, diabetes treatment
BMI	Sex, Smoking
Total Cholesterol	Sex, BMI, smoking, Lipid lowering treatment
HDL-cholesterol	Sex, BMI, smoking, Lipid lowering treatment
Triacylglycerols	Sex, BMI, Smoking, Lipid lowering treatment

ESM Table 2: Baseline characteristics stratified by age group and sex.

Variable	Overall Men	Overall Women	18-44 Men	18-44 Women	45-59 Men	45-59 Women	60-74 Men	60-74 Women	75- Men	75- Women
n	55681	44925	5200	3442	19212	11695	25404	19865	5865	9923
Follow-up time (years)	2.9 (2.5)	2.8 (2.5)	2.6 (2.5)	2.4 (2.4)	2.9 (2.6)	3.0 (2.6)	2.9 (2.5)	2.8 (2.5)	3.0 (2.4)	2.6 (2.3)
Women n (%)	0 (0.0%)	44925 (100.0%)	0 (0.0%)	3442 (100.0%)	0 (0.0%)	11695 (100.0%)	0 (0.0%)	19865 (100.0%)	0 (0.0%)	9923 (100.0%)
Age (years)	60.4 (11.7)	64.0 (12.9)	38.7 (5.4)	37.8 (5.9)	53.1 (4.2)	53.2 (4.2)	65.9 (4.0)	66.8 (4.2)	80.4 (4.4)	80.4 (4.4)
HbA_{1c} (mmol/mol)	49.6 (11.5)	48.7 (10.3)	52.5 (15.5)	50.1 (12.9)	50.8 (12.6)	49.8 (11.6)	48.5 (10.0)	47.9 (9.3)	48.6 (9.4)	48.5 (9.4)
$HbA_{1c\ (\%)}$	6.7 (1.1)	6.6 (0.9)	7.0 (1.4)	6.7 (1.2)	6.8 (1.2)	6.7 (1.1)	6.6 (0.9)	6.5 (0.9)	6.6 (0.9)	6.6 (0.9)
HbA_{1c} > 53 mmol/mol n (%)	9806 (22.5%)	6656 (19.2%)	1178 (31.1%)	604 (24.8%)	3877 (26.1%)	2124 (23.2%)	3800 (19.0%)	2560 (16.5%)	951 (19.6%)	1368 (18.1%)
BMI (kg/m2)	30.2 (5.2)	31.0 (6.2)	32.7 (6.8)	34.5 (7.9)	31.0 (5.3)	32.3 (6.4)	29.7 (4.6)	30.8 (5.8)	27.5 (4.0)	28.8 (5.0)
BMI > 30 kg/m2 n (%)	19013 (34.1%)	17424 (38.8%)	2348 (45.2%)	1783 (51.8%)	7587 (39.5%)	5377 (46.0%)	8066 (31.8%)	7687 (38.7%)	1012 (17.3%)	2577 (26.0%)
Systolic BP (mmHg)	136.9 (16.9)	137.9 (17.7)	130.1 (15.2)	125.7 (15.1)	135.1 (16.4)	134.5 (16.6)	138.7 (16.9)	139.5 (17.0)	140.1 (18.0)	142.5 (18.4)
Systolic hypertension (>140 mmHg) n (%)	14257 (25.6%)	12509 (27.8%)	664 (12.8%)	318 (9.2%)	4255 (22.1%)	2482 (21.2%)	7414 (29.2%)	6088 (30.6%)	1924 (32.8%)	3621 (36.5%)
Diastolic BP (mmHg)	80.6 (10.1)	78.6 (9.8)	81.4 (10.6)	79.1 (10.2)	82.7 (10.0)	81.0 (9.7)	80.1 (9.6)	78.6 (9.4)	75.5 (9.7)	75.7 (9.8)
Diastolic hypertension (>90 mmHg) n (%)	5149 (9.2%)	2769 (6.2%)	570 (11.0%)	239 (6.9%)	2407 (12.5%)	1087 (9.3%)	1989 (7.8%)	1120 (5.6%)	183 (3.1%)	323 (3.3%)

Variable	Overall Men	Overall Women	18-44 Men	18-44 Women	45-59 Men	45-59 Women	60-74 Men	60-74 Women	75- Men	75- Women
Total cholesterol (mmol/L)	5.1 (1.2)	5.4 (1.2)	5.3 (1.3)	5.2 (1.1)	5.3 (1.2)	5.5 (1.2)	5.1 (1.1)	5.4 (1.2)	4.8 (1.1)	5.3 (1.1)
Hypercholesterolaemia (>6.2 mmol/L) n (%)	5998 (10.8%)	6210 (13.8%)	641 (12.3%)	298 (8.7%)	2579 (13.4%)	1822 (15.6%)	2459 (9.7%)	3060 (15.4%)	319 (5.4%)	1030 (10.4%)
LDL cholesterol (mmol/L)	3.1 (1.0)	3.2 (1.0)	3.2 (1.0)	3.2 (0.9)	3.2 (1.0)	3.3 (1.0)	3.0 (1.0)	3.2 (1.0)	2.8 (0.9)	3.0 (1.0)
HDL cholesterol (mmol/L)	1.2 (0.4)	1.3 (0.4)	1.0 (0.3)	1.1 (0.3)	1.1 (0.3)	1.3 (0.4)	1.2 (0.4)	1.4 (0.4)	1.2 (0.4)	1.4 (0.4)
Triacylglycerols (mmol/L)	2.1 (1.5)	1.9 (1.1)	2.6 (2.0)	2.0 (1.4)	2.4 (1.8)	2.0 (1.3)	2.0 (1.3)	1.9 (1.0)	1.7 (0.9)	1.8 (0.8)
Microalbuminuria n (%)	3830 (13.8%)	2280 (10.5%)	316 (12.1%)	176 (10.7%)	1214 (12.3%)	606 (10.4%)	1765 (14.0%)	967 (9.8%)	535 (19.6%)	531 (12.3%)
eGFR (mL/min)	88.4 (25.3)	80.2 (24.2)	106.7 (27.1)	104.4 (26.6)	95.3 (26.1)	90.6 (23.6)	84.4 (21.0)	78.4 (20.8)	69.8 (21.2)	65.3 (18.8)
Smokers n (%)	8162 (18.9%)	6095 (17.3%)	948 (24.6%)	592 (23.4%)	3688 (24.6%)	2429 (26.3%)	3232 (16.3%)	2643 (16.8%)	294 (6.5%)	431 (5.6%)
Antihypertensives n (%)	61298 (59.7%)	54074 (65.3%)	2242 (23.9%)	1360 (21.9%)	17844 (50.6%)	11154 (52.2%)	32888 (69.7%)	26372 (71.6%)	8324 (76.6%)	15188 (82.5%)
Statins n (%)	40484 (39.3%)	30680 (37.2%)	1808 (19.0%)	762 (12.2%)	12468 (35.1%)	6682 (31.0%)	21742 (46.1%)	16316 (44.5%)	4466 (41.4%)	6920 (38.4%)
No medication	27554 (49.5%)	24937 (55.5%)	2112 (40.6%)	1712 (49.7%)	8371 (43.6%)	5723 (48.9%)	13360 (52.6%)	11173 (56.2%)	3711 (63.3%)	6329 (63.8%)
Oral agents	22284 (40.0%)	16298 (36.3%)	2491 (47.9%)	1482 (43.1%)	8453 (44.0%)	4744 (40.6%)	9728 (38.3%)	7166 (36.1%)	1612 (27.5%)	2906 (29.3%)
Insulin	3395 (6.1%)	2194 (4.9%)	375 (7.2%)	150 (4.4%)	1319 (6.9%)	712 (6.1%)	1324 (5.2%)	870 (4.4%)	377 (6.4%)	462 (4.7%)

Variable	Overall Men	Overall Women	18-44 Men	18-44 Women	45-59 Men	45-59 Women	60-74 Men	60-74 Women	75- Men	75- Women
Insulin + Oral agents	2448 (4.4%)	1496 (3.3%)	222 (4.3%)	98 (2.8%)	1069 (5.6%)	516 (4.4%)	992 (3.9%)	656 (3.3%)	165 (2.8%)	226 (2.3%)

All data are based on the first observation after diagnosis, except for HbA1c where the first observation 1 year after diagnosis was used' All continuous variables are represented as mean with standard deviation in parentheses. Dichotomous variables are represented as number of individuals with percentage in parentheses. BMI = body mass index, BP = blood pressure, eGFR = estimated glomerular filtration rate, HbA1c = glycated haemoglobin, HDL = high-density lipoprotein, LDL = low-density lipoprotein, n = number of individuals. Initial medication data were obtained within 3 months of diagnosis. RoW = rest of world.

ESM figure 1

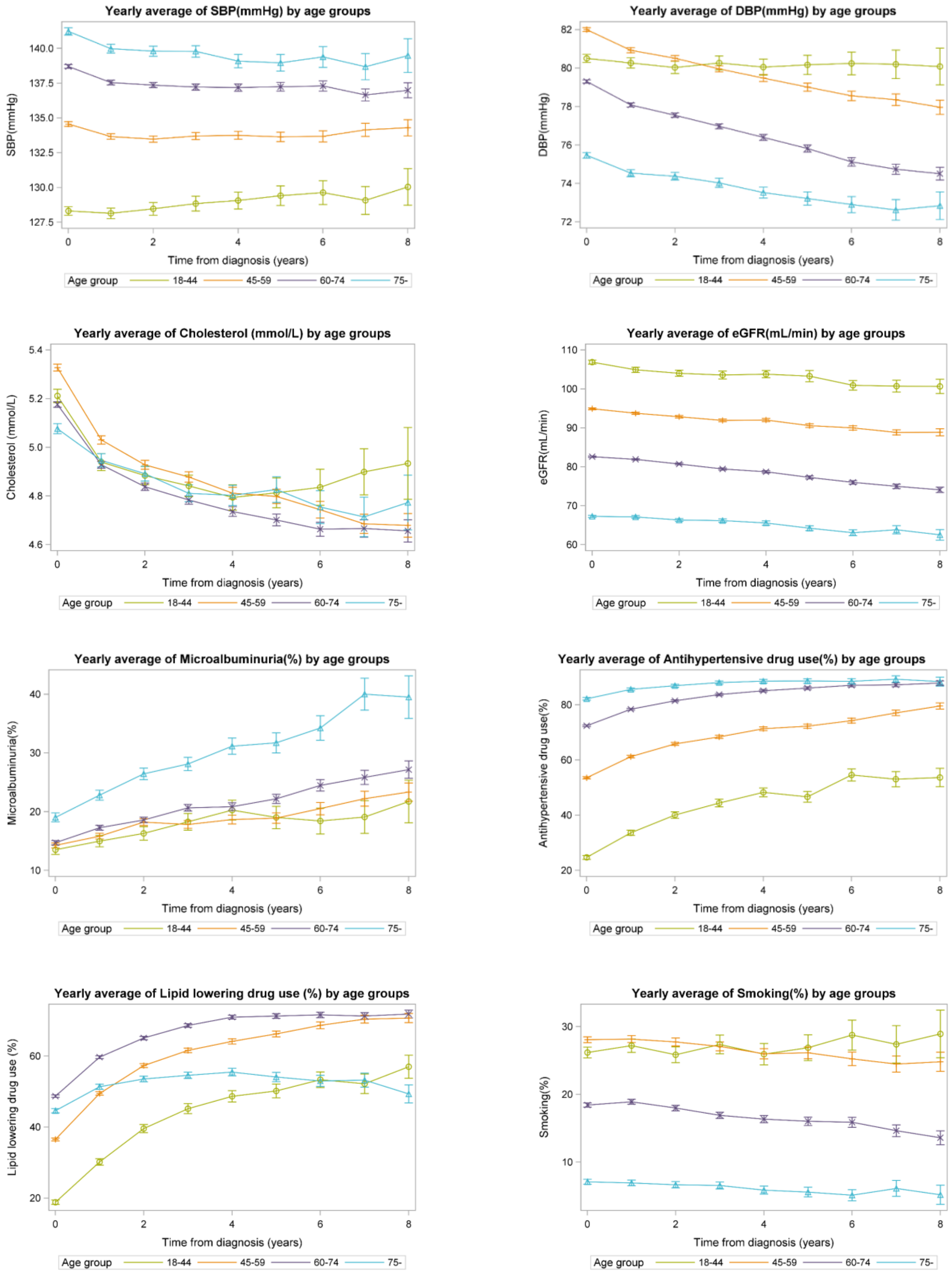

Figures 1a-h. 1a = systolic blood pressure (SBP); 1b = diastolic blood pressure (DBP); 1c = total cholesterol; 1d = estimated glomerular filtration rate (eGFR); 1e = percentages of microalbuminuria; 1f = percentages of antihypertensive drug use; 1g = lipid lowering drug use; 1h = smoking.

ESM Figure 2

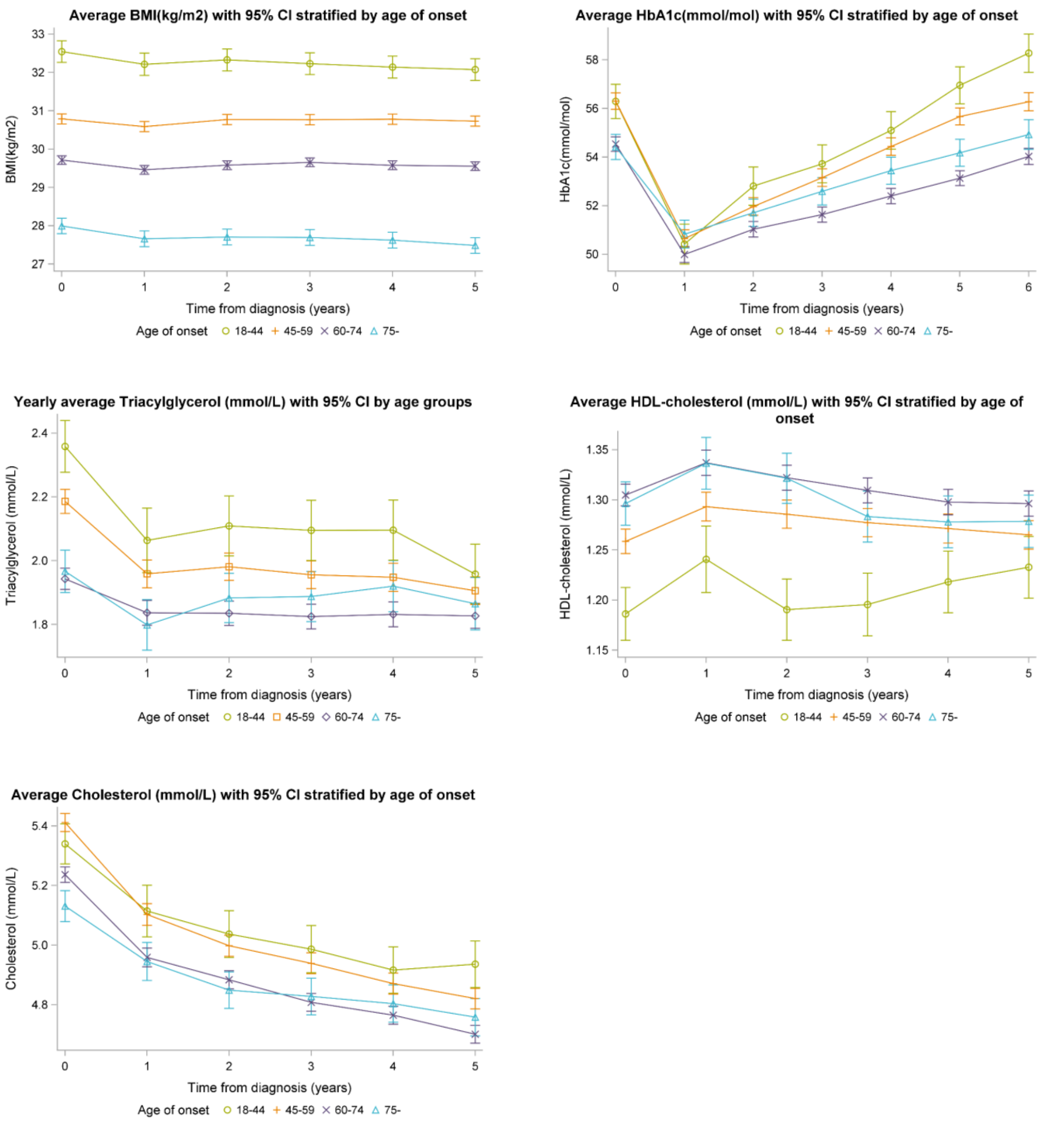

Figures 2a-e: 2a = BMI for those with at least 5 year follow-up data. 2b = HbA_{1c} for those with at least 5 year follow-up data after nadir at year 1; 2c = Triacylglycerol for those with at least 5 year follow-up data; 2d = HDL-c for those with at least 5 year follow-up data; 2e = total cholesterol for those with at least 5 year follow-up data. *In each case, data also adjusted for confounders in ESM Table 1 above.*

ESM Figure 3

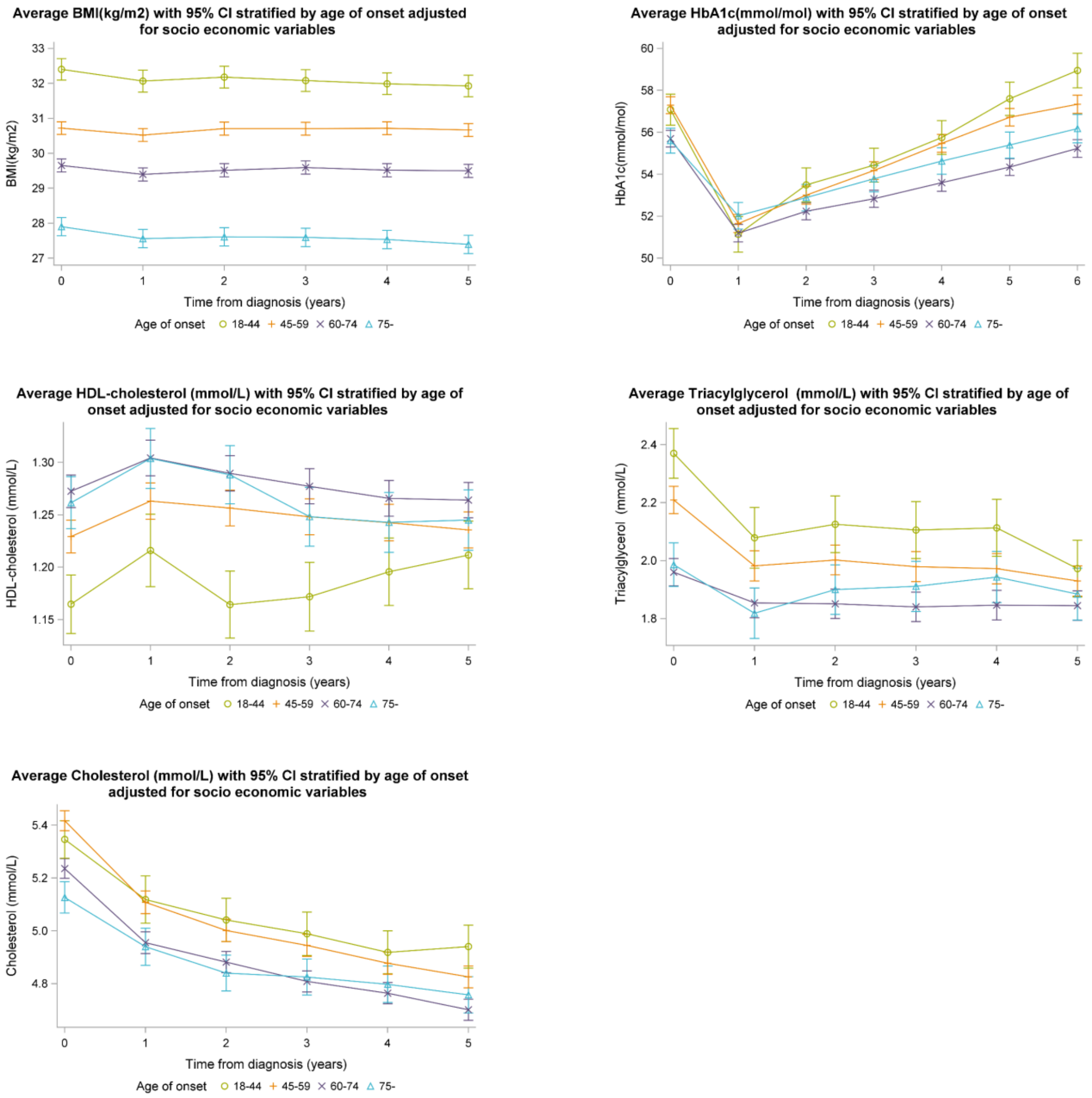

Figures 3a-e: 3a = BMI stratified by age group for those with at least 5 year follow-up data; 3b = HbA_{1c} for those with at least 5 year follow-up data after nadir at year 1; 3c = HDL-c for those with at least 5 year follow-up data; 3d = triacylglycerol for those with at least 5 year follow-up data; 3e = cholesterol for those with at least 5-year follow-up data. *In each case, data also adjusted for confounders in ESM Table 1 and in addition for marital status, education and country of birth*.
